# The Effect of Electroacupuncture on PKMzeta in the ACC in Regulating Anxiety-Like Behaviors in Rats Experiencing Chronic Inflammatory Pain

**DOI:** 10.1155/2017/3728752

**Published:** 2017-09-18

**Authors:** Junying Du, Junfan Fang, Cun Wen, Xiaomei Shao, Yi Liang, Jianqiao Fang

**Affiliations:** Department of Neurobiology and Acupuncture Research, The Third Clinical Medical College, Zhejiang Chinese Medical University, Hangzhou 310053, China

## Abstract

Chronic inflammatory pain can induce emotional diseases. Electroacupuncture (EA) has effects on chronic pain and pain-related anxiety. Protein kinase Mzeta (PKMzeta) has been proposed to be essential for the maintenance of pain and may interact with GluR1 to maintain CNS plasticity in the anterior cingulate cortex (ACC). We hypothesized that the PKMzeta-GluR1 pathway in the ACC may be involved in anxiety-like behaviors of chronic inflammatory pain and that the mechanism of EA regulation of pain emotion may involve the PKMzeta pathway in the ACC. Our results showed that chronic inflammatory pain model decreased the paw withdrawal threshold (PWT) and increased anxiety-like behaviors. The protein expression of PKCzeta, p-PKCzeta (T560), PKMzeta, p-PKMzeta (T560), and GluR1 in the ACC of the model group were remarkably enhanced. EA increased PWT and alleviated anxiety-like behaviors. EA significantly inhibited the protein expression of p-PKMzeta (T560) in the ACC, and only a downward trend effect for other substances. Further, the microinjection of ZIP remarkably reversed PWT and anxiety-like behaviors. The present study provides direct evidence that the PKCzeta/PKMzeta-GluR1 pathway is related to pain and pain-induced anxiety-like behaviors. EA treatment both increases pain-related somatosensory behavior and decreases pain-induced anxiety-like behaviors by suppressing PKMzeta activity in the ACC.

## 1. Introduction

Pain has both sensory-discriminative and emotional-affective dimensions. It has been known that a large proportion of chronic pain patients have accompanying sleep, depressive, and/or anxiety disorders that contribute to a deterioration in the quality of life [1, 2]. Patients with chronic pain are up to 2-3 times more likely to develop anxiety disorders [3, 4]. Chronic inflammatory pain, which is one of the most common chronic pain conditions, is strongly associated with psychiatric disorders (e.g., anxiety and depressive disorders) [5]. Although the mechanisms underlying the comorbidity of chronic inflammatory pain and anxiety have not been defined, preclinical studies have demonstrated a relationship between chronic inflammatory pain and mood disorders in animal models. In fact, previous studies by our lab and others have shown that inducing chronic inflammatory pain for four weeks using complete Freund's adjuvant (CFA) results in the development of anxiety-like behaviors [6–8].

Electroacupuncture (EA), which is a modern method of administering acupuncture, involves the application of a pulsating electrical current to acupuncture needles for acupuncture point stimulation. EA has been used to treat a variety of diseases and symptoms, including pain. Recent studies, including our own, have shown that EA can increase the paw withdrawal threshold (PWT) in inflammatory pain and neuropathic pain induced by noxious stimulation [9–12]. In recent years, the mechanism and treatment of psychiatric disorders associated with chronic pain have received increasing attention. Previous studies by our laboratory and others have reported that EA can significantly reduce the anxiety-like behaviors associated with chronic pain [13, 14]. However, the mechanism for these effects is unclear.

A large body of evidence, including both clinical and experimental research, indicates that the anterior cingulate cortex (ACC) plays a role in the processing of noxious stimuli and in pain-related depression and anxiety [15–22]. Protein kinase Mzeta (PKMzeta), an isoform of protein kinase C with persistent activity, is involved in the neural plasticity associated with pain and anxiety disorders [23, 24]. Li et al. have shown that PKMzeta in the ACC acts to maintain neuropathic pain. PKMzeta may thus be a novel therapeutic target for treating chronic pain [25]. It has also been shown that CFA-induced mechanical and thermal hypersensitivity is reduced by the inhibition of PKCzeta/PKMzeta activity [26]. Another study demonstrated that PKMzeta may interact with GluR1 to maintain long-term potentiation (LTP) in the ACC [27] and hippocampus [28].

We hypothesized that the PKMzeta-GluR1 pathway in the ACC may be involved in anxiety-like behaviors in rats experiencing chronic inflammatory pain and that the mechanism of EA regulation of pain-related emotion may involve the PKMzeta pathway in the ACC. To test these hypotheses, we first used a rat CFA model to determine whether the PKMzeta pathway plays an important role in anxiety-like behaviors. We then investigated whether EA can relieve anxiety-like behaviors by counteracting activation of the PKMzeta pathway.

## 2. Materials and Methods

### 2.1. Animals

Adult male Sprague-Dawley (SD) rats (220–250 g) were purchased through the Shanghai Laboratory Animal Center, Chinese Academy of Sciences (Animal Certificate number SCXK (沪) 2013-0016). Rats were housed six per cage in a controlled environment (temperature: 25 ± 2°C, humidity: 55% ± 5%, and light: 12 h light/dark cycle) and were fed a standard rodent food and allowed distilled water ad libitum. Rats were given at least 1 week to adapt to the new environment before any manipulation. All experimental procedures were approved by the Animal Ethics Committee of Zhejiang Chinese Medical University (ZSLL-2015-022).

### 2.2. Experimental Design

Two sets of experiments were conducted in this paper. In experiment 1, animals were randomly divided into four groups: (1) control group, (2) model group, (3) EA group, and (4) sham EA group. In experiment 2, the animals that were implanted with a guide cannula for microinjection were randomly divided into two groups: (1) CFA + saline group and (2) CFA + zeta-interacting protein (ZIP) group.

### 2.3. Induction of Inflammatory Pain

Inflammatory pain was induced with an injection of 100 *μ*l of complete Freund's adjuvant (CFA, Sigma, USA) into the plantar surface of the right hind paw of SD rats.

### 2.4. Mechanical Hyperalgesia Behavioral Testing

The paw withdrawal threshold (PWT), which indicates mechanical hyperalgesia, was determined using the up-down method of Chaplan et al. [29]. The paw withdrawal threshold was measured before CFA injection (as a base), 1 day after CFA injection (before EA stimulation), and 28 days after CFA injection (after EA stimulation). The paw withdrawal threshold was measured between 9:00 am and 17:00 pm. Rats were acclimated to the test environment in individual testing cages for 30 min each day for three consecutive days. On the test day, rats were given at least 20 min to acclimate. The von Frey hairs (Stoelting Co., Thermo, USA) were applied in a consecutive ascending order (0.4, 0.6, 1, 2, 4, 6, 8, 15, and 26 g) perpendicularly to the central surface of the hind paw and held for approximately 6–8 s to determine the paw withdrawal threshold. The first hair applied corresponded to a force of 4 g. In the absence of a paw withdrawal response to the initially selected hair, expressed as O, a stronger stimulus was presented; in the event of paw withdrawal, expressed as X, the next weaker stimulus was chosen. This method requires 6 response data points. Counting of the critical 6 data points did not begin until the response threshold was first crossed, at which time the 2 responses straddling the threshold were retrospectively designated the first 2 responses in the series of 6. In cases where continuous positive or negative responses were observed to the exhaustion of the stimulus set, values of 26 g and 0.4 g were assigned, respectively. The paw withdrawal threshold was interpolated using the following formula: paw withdrawal threshold = (10^[Xf + *κδ*])/10000, where Xf = the value (in log units) of the final von Frey hair used; *κ* = the tabular value for the pattern of positive/negative responses; and *δ* = the mean difference (in log units) between stimuli (here, 0.231).

### 2.5. Anxiety-Like Behavioral Tests

Anxiety-like behaviors were quantified using the elevated zero maze test and the open-field test. All animals were acclimatized twice to the experimental conditions (temperature 22 ± 3°C, ambient noise < 40 dB) before testing. All external lights were obscured. Smart 3.0 software (Panlab, USA) was used to image acquisition and data analysis. The testing platforms were cleaned with 10% alcohol between animals, and individual tests were separated by 3-min intervals.

#### 2.5.1. Open-Field Test

The open-field test consists of a Plexiglas arena (100 cm × 100 cm × 50 cm). After animals were habituated to the experimental conditions, they were placed in the center of the arena and their exploratory behaviors were recorded for 10 min. Smart 3.0 software (Panlab, USA) was used to analyze the video. The arena was divided into 16 squares, 4 of which comprise the central zone, while the others comprise the peripheral zone. The parameters measured included the number of entries into the central zone, the percentage of time in the central zone, the distance in the central zone, and the total distance traveled throughout the arena.

#### 2.5.2. Elevated Zero Maze

The elevated zero maze consists of a black metallic annular platform (100 cm in diameter, 25 cm in width, and 55 cm in height) divided equally into four quadrants. There are two closed arms (opposing each other) flanked by 30-cm-high black metallic walls, while the remaining two arms are without walls (open arms). After the animals were habituated to the experimental conditions, they were placed in the place between the open arm and closed arm facing the open arm, and their exploratory behaviors were recorded for 5 min. Smart 3.0 software (Panlab, USA) was used to analyze the video. The parameters measured included the number of entries into the open arm, the time in the open arm, the distance traveled in the open arm, and the total distance traveled throughout the arms.

### 2.6. Electroacupuncture Stimulation

Rats in the EA and sham EA groups were gently immobilized using a special cotton retainer designed by our laboratory (Patent number ZL 2014 2 0473579.9, State Intellectual Property Office of the People's Republic of China). Stainless steel acupuncture needles were inserted to a depth of 1 cm at ST36 (Zusanli) and BL60 (Kunlun) bilaterally in rats in the EA group. The needles were connected to a HANS Acupuncture Point Nerve Stimulator (Hans-100, Huawei Co., Ltd., Beijing, China), and the output parameters were set as follows: 2 and 100 Hz alternating frequencies (automatically shifting between 2 and 100 Hz stimulation for 3 s each) and intensities ranging from 0.5 to 1.5 mA (10 min each, total 30 min). EA stimulation was administered once daily for 30 min beginning on day 1. EA stimulation was performed on days 28–30 after CFA injection.

### 2.7. Implantation of a Guide Cannula for Microinjection

Rats (weighing 250–280 g) in the CFA + saline group and CFA + ZIP group were anesthetized with isoflurane using a ventilator. A guide cannula (OD 0.64 mm–23 G/m, 900-0062-001, RWD, China) was implanted into the ipsilateral ACC for intracerebroventricular microinjection. The ACC (Paxinos-Watson) coordinate was 1.5 mm anterior to the bregma, 0.3 mm lateral to the midline, and 1.8 mm ventral to the surface of the endocranium. The cannulas were anchored to the skull with stainless-steel screws and dental cement. A stainless-steel stylet blocker was inserted into each cannula to keep them patent and to prevent infection. The rats were allowed to recover for 7 days after surgery. For microinjection, the rats were restrained in a special cotton retainer (Patent number ZL 2014 2 0473579.9, State Intellectual Property Office of the People's Republic of China), and a small hole was made over the guide cannulas. A single injector (OD 0.41 mm–27 G/Mates with M3.5, 900-0062-201, RWD, China) was inserted into the guide cannula with fixed screw-single (OD 5.5 mm/l, 7.5 mm/M3.5, 900-0062-501, RWD, China) and then connected to Teflon tuble (JT-10, 809210, EICOM CORP, Japan). The microinjection was conducted using a microsyringe pump (Micro 4™, WPI, USA) and a microsyringe (WPI, USA). Rats in the CFA + ZIP group were injected with ZIP (539624, Millipore, USA) dissolved in 0.9% NS (30 nmol/*μ*l, 1 *μ*l over 1 min) into the ipsilateral ACC once daily from day 28 to day 30. Rats in the CFA + saline group were injected with 0.9% NS (1 *μ*l over 1 min) into the ipsilateral ACC once daily from day 28 to day 30.

### 2.8. Western Blotting Analysis

After behavioral testing, rats were administered 10% choral hydrate i.p. in a volume of 0.35 ml per 100 g bodyweight. Once the animals were deeply anesthetized, they were perfused with 200 ml of ice-cold saline to clear contaminating blood. The ACC (AP: +4.4 mm–1.4 mm, ML: +0.6 mm, and DV: 3 mm, Paxinos-Watson) was quickly removed and stored at −80°C. The samples were added to Western & IP buffer (20 mM Tris (pH 7.5), 150 mM NaCl, 1% Triton X-100 sodium, pyrophosphate, *β*-glycerophosphate, EDTA, Na3VO4, leupeptin, etc., P0013, Beyotime, China) containing PMSF (ST505, Beyotime, China) and protease/phosphatase inhibitor cocktail (P1260, Applygen, China) and homogenized using ultrasonic agitation. The homogenate was allowed to rest on ice for 30 min and was then centrifuged at 15,000 rcf for 15 min at 4°C, and the supernatant was collected. The protein concentration of the tissue lysates was determined with a BCA Protein Assay Kit. 20 *μ*g of protein was loaded into each lane and separated on 5%–10% SDS-PAGE gel. Then the protein was electrophoretically transferred to polyvinylidene fluoride (PVDF) membranes (0.45 *μ*m, Bio-Rad, USA). The membranes were blocked with 5% low-fat milk in TBST for 1 h at room temperature and then incubated overnight with the following antibodies: rabbit anti-rat PKCzeta (1 : 1000, Abcam, USA), rabbit anti-rat p-PKCzeta (phospho T560, 1 : 1000, Abcam, USA), and horseradish peroxidase- (HRP-) conjugated rabbit anti-rat GAPDH (1 : 1000, Cell Signaling, USA). The membranes were incubated for 1 h at room temperature in HRP-conjugated goat anti-rabbit IgG as the secondary antibody (1 : 10000, Abcam, USA). The membranes were developed with an ECL kit (Beyotime, China), and the signals were captured with Image Quant LAS 4000 (GE, USA). The scanned images were analyzed by Image Quant TL7.0 Analysis Software (GE, USA).

### 2.9. Statistical Analysis

All data are expressed as the mean ± standard error mean. The paw withdrawal threshold data were analyzed using a repeated-measures ANOVA with between-subject factors. All other experiments were analyzed using a one-way ANOVA. Post hoc testing was performed using Fisher's PLSD test to detect differences between the groups. The criterion for statistical significance was *P* < 0.05.

## 3. Results

### 3.1. The Effect of EA on CFA-Induced Allodynia

**Figure 1:**
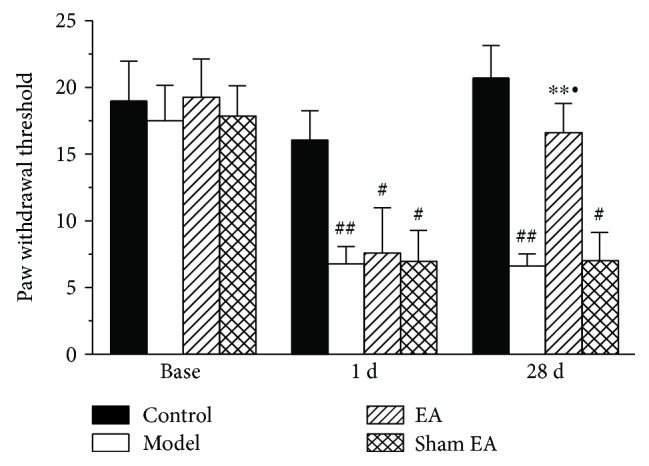
The effect of EA on the paw withdrawal threshold. The paw withdrawal threshold was tested at base, 1 day, and 28 days after CFA injection. The paw withdrawal threshold was decreased from day 1 to day 28 after CFA injection. EA stimulation was initiated on day 28 and significantly increased the paw withdrawal threshold. All data represent the mean ± SEM, *n* = 8. ^#^*P* < 0.05 and ^##^*P* < 0.01, compared to the control group; ^∗∗^*P* < 0.01, compared to the model group; ^•^*P* < 0.05, compared to the sham EA group.

CFA is a commonly used model of chronic pain that induces prolonged allodynia. To determine whether EA stimulation could reduce the allodynia induced by this agent, rats received an intraplantar injection of CFA to induce peripheral chronic pain and received EA stimulation for 30 min on the day 28 following the CFA injection. As shown in Figure 1, before CFA injection, the PWT (base) on the ipsilateral paw was not significantly different between groups (*P* > 0.05). The PWT on the ipsilateral paw was remarkably reduced 1 day after CFA injection, and this effect lasted for 28 days (*P* < 0.01). EA stimulation was initiated on day 28 after CFA injection. A single application of EA was effective to increase the PWT on the ipsilateral paws (*P* < 0.05). Conversely, sham EA stimulation did not increase the PWT (*P* > 0.05).

### 3.2. The Effect of EA on Anxiety-Like Behaviors Induced by Chronic Pain

We next investigated whether EA could alleviate the anxiety-like behaviors induced by chronic inflammatory pain. In this study, rats were tested for anxiety-like behavior on days 29 and 30 after pain induction using the open-field test and elevated zero maze, respectively.

**Figure 2:**
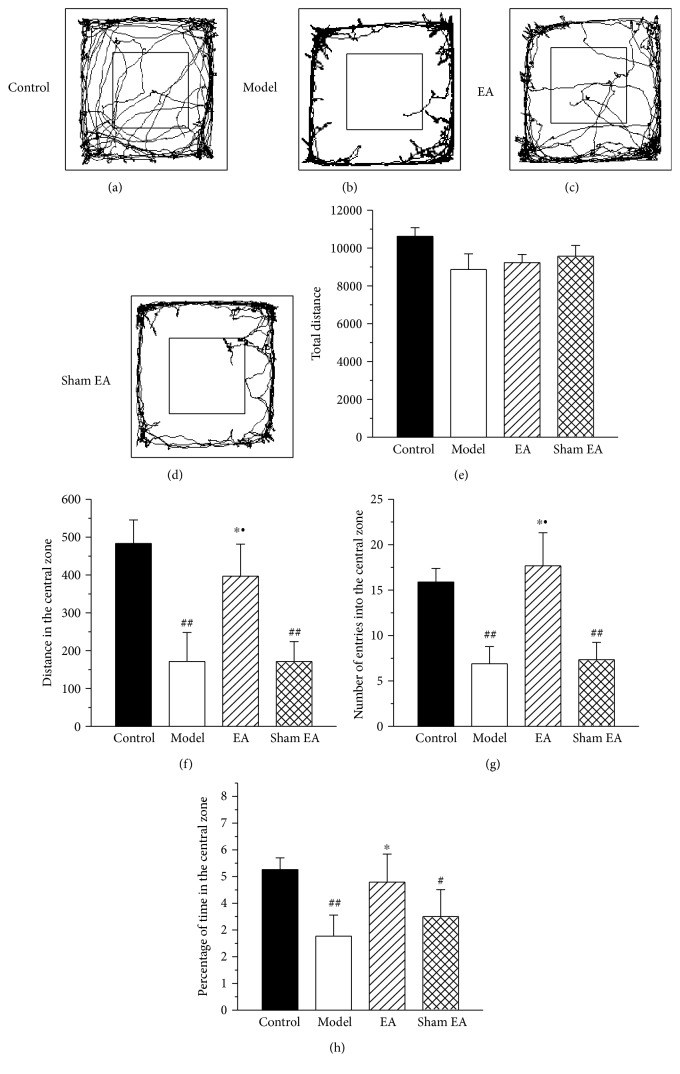
The effect of EA on chronic inflammatory pain-induced anxiety-like behaviors in open-field test. (a–d) The trajectories of rats in the control group, model group, EA group, and sham EA group in the open-field test on day 29 after CFA injection. (e–h) The total distance traveled throughout the arena (e), distance in the central zone (f), number of entries into the central zone (g), and percentage of time in the central zone (h) of the control group, model group, EA group, and sham EA group. All data represent the mean ± SEM, *n* = 8. ^#^*P* < 0.05 and ^##^*P* < 0.01, compared to the control group; ^∗^*P* < 0.05, compared to the model group; ^•^*P* < 0.05, compared to the sham EA group.

In the open-field test, which was administered on day 29 after CFA injection, the model rats displayed increased anxiety-like behavior, compared to the control rats (Figure 2). Compared to the control rats, the model rats traveled significantly less distance in the central zone, made fewer number of entries into the central zone, and spent a reduced percentage of time in the central zone (Figures 2(f), 2(g), and 2(h)). The locomotion of the model rats was unchanged compared to that of the control rats, as indicated by the total distance in the open field (Figure 2(e)). EA stimulation remarkably increased the distance in the central zone, the number of entries into the central zone, and the percentage of time in the central zone, whereas sham EA did not affect these parameters (Figures 2(f), 2(g), and 2(h)).

**Figure 3:**
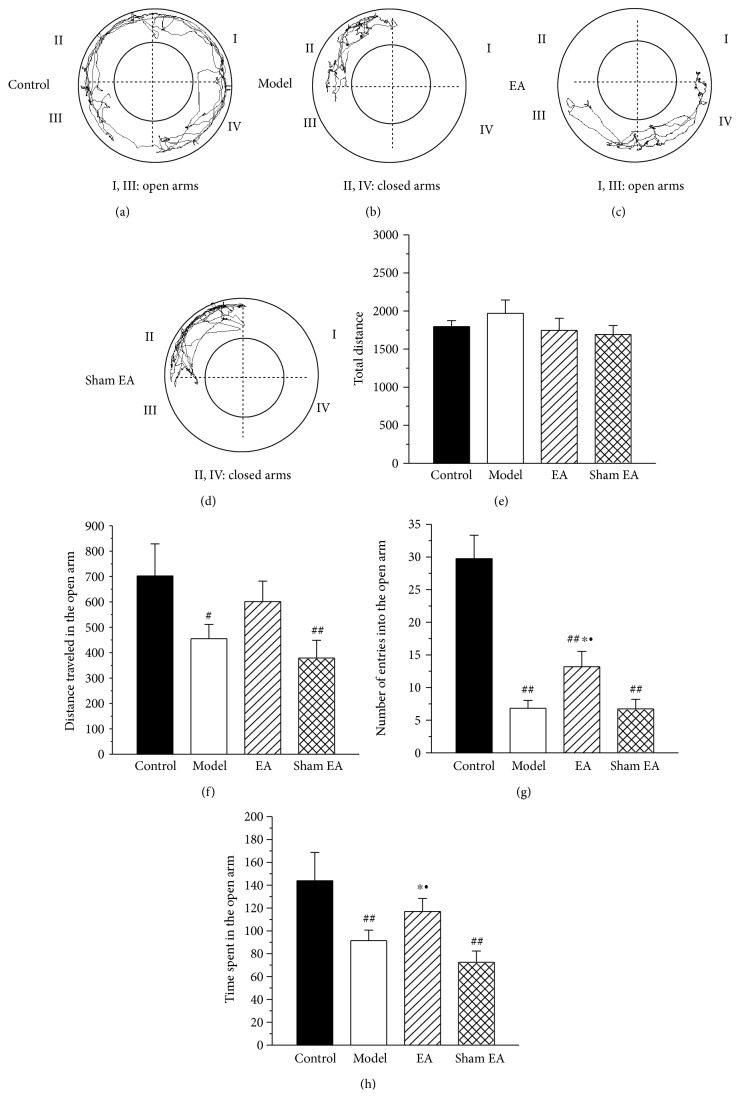
The effect of EA on chronic inflammatory pain-induced anxiety-like behaviors in elevated zero maze. (a–d) The trajectories of rats in the control group, model group, EA group, and sham EA group in the elevated zero maze on day 30 after CFA injection. Total distance traveled throughout the arms (e), distance traveled in the open arm (f), the number of entries into the open arm (g), and time spent in the open arm (h) of the control group, model group, EA group, and sham EA group. All data represent the mean ± SEM, *n* = 8. ^#^*P* < 0.05 and ^##^*P* < 0.01, compared to the control group; ^∗^*P* < 0.05, compared to the model group; ^•^*P* < 0.05, compared to the sham EA group.

In the elevated zero maze, which was administered on day 30 after the CFA injection, the model rats exhibited increased anxiety-like behavior, compared to the control rats (Figure 3). Compared to the control rats, the model rats traveled less distance in the open arm, had a reduced number of entries into the open arm, and spent less time in the open arm (Figures 3(f), 3(g), and 3(h)). There was no difference in the total distance traveled between the control rats and the model rats (Figure 3(e)). EA stimulation remarkably increased the number of entries into the open arm and the time in the open arm. In contrast, sham EA did not affect these parameters (Figures 3(f), 3(g), and 3(h)).

### 3.3. The Effect of EA on the Expression of PKCzeta and p-PKCzeta (T560) Protein in the ACC

**Figure 4:**
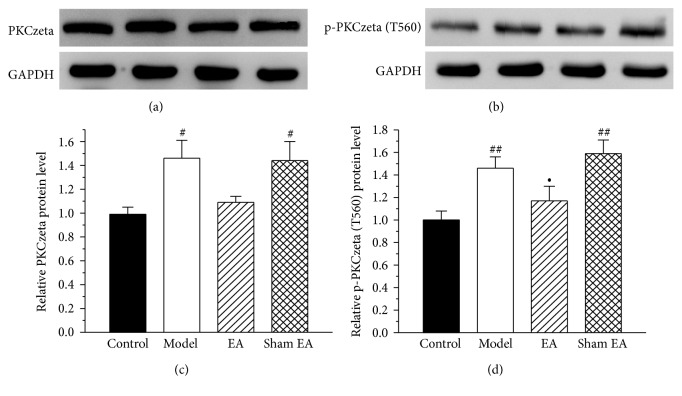
The effect of EA on PKCzeta and p-PKCzeta (T560) protein expression in the ACC. (a, b) Western blotting analysis revealed that EA showed a trend reduce the protein expression of PKCzeta and p-PKCzeta (T560) proteins on day 30 after CFA injection. (c) Quantification of (a) PKCzeta protein normalized to the level of GAPDH. (d) Quantification of (b) p-PKCzeta (T560) protein normalized to the level of GAPDH. All data represent the mean ± SEM, *n* = 6. ^#^*P* < 0.05 and ^##^*P* < 0.01, compared to the control group; ^•^*P* < 0.05, compared to the sham EA group.

To investigate a possible increase in the expression of PKCzeta and p-PKCzeta (T560) protein in the ACC following CFA injection and its modulation by EA stimulation, we evaluated the expression of these proteins in the ACC in the control group, the model group, the EA group, and the sham EA group on day 30 after CFA injection. As shown in Figure 4, compared to the control group, the level of PKCzeta and p-PKCzata (T560) protein in the ACC of the model group was significantly increased (*P* < 0.05). The protein levels were not increased in the EA group. Meanwhile, EA stimulation showed a trend to reduce the PKCzeta and p-PKCzeta (T560) protein, and there was no significant difference between the model group and the EA group (*P* > 0.05).

### 3.4. The Effect of EA on the Expression of PKMzeta and p-PKMzeta (T560) Protein in the ACC

**Figure 5:**
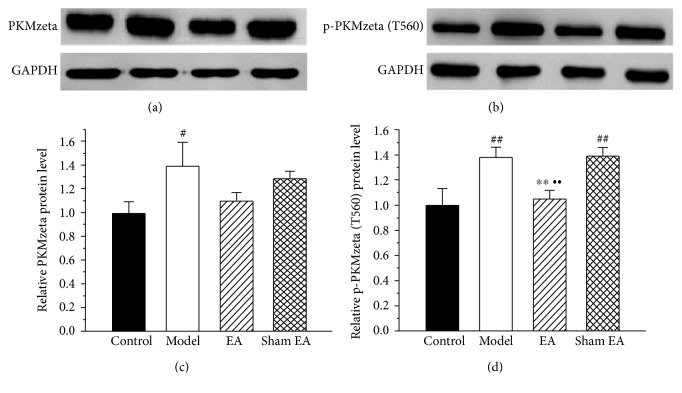
The effect of EA on PKMzeta and p-PKMzeta (T560) protein expression in the ACC. (a, b) Western blotting analysis revealed that EA significantly reduced the protein expression of p-PKMzeta (T560) on day 30 after CFA injection, but not PKMzeta, in the ACC. (c) Quantification of (a) PKMzeta protein normalized to the level of GAPDH. (d) Quantification of (b) p-PKMzeta (T560) protein normalized to the level of GAPDH. All data represent the mean ± SEM, *n* = 6. ^#^*P* < 0.05 and ^##^*P* < 0.01, compared to the control group; ^∗∗^*P* < 0.01, compared to the model group; ^••^*P* < 0.01, compared to the sham EA group.

To investigate the effect PKMzeta (51KDa) protein, a smaller fragment of PKCzeta (75KDa), in the chronic inflammatory model and its potential modulation by EA, we next evaluated the expression of PKMzeta in the ACC of the control group, the model group, the EA group, and the sham EA group on day 30 after CFA injection. As shown in Figure 5, the expression of PKMzeta and p-PKMzeta (T560) proteins in the ACC of the model group was increased compared to that of the control group (*P* < 0.05). After EA stimulation, a trend toward a decrease in PKMzeta protein was observed for rats with EA stimulation, although this change was not statistically significant (Figure 5(c)). In addition, EA stimulation significantly reduced the overexpression of p-PKMzeta (T560) in the ACC (*P* < 0.05) (Figure 5(d)). In contrast, sham EA stimulation failed to reduce either PKMzeta or p-PKMzeta (T560) protein expression (*P* > 0.05) (Figures 5(c) and 5(d)).

### 3.5. The Effect of EA on the Expression of GluR1 Protein in the ACC

**Figure 6:**
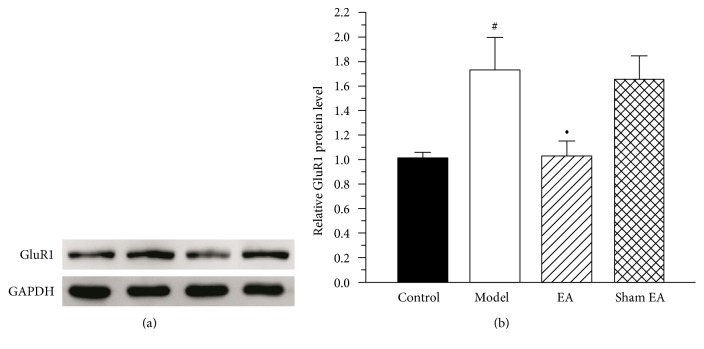
The effect of EA on GluR1 protein expression in the ACC. (a) Western blotting analysis revealed that EA showed a trend to reduce the GluR1 protein expression on day 30 after CFA injection. (b) Quantification of (a) GluR1 protein normalized to the level of GAPDH. All data represent the mean ± SEM, *n* = 6. ^#^*P* < 0.05, compared to the control group; ^•^*P* < 0.05, compared to the sham EA group.

GluR1 is a downstream target of PKMzeta; thus, we also measured the expression of GluR1 protein in the ACC of the control group, the model group, the EA group, and the sham EA group on day 30 after CFA injection. As shown in Figure 6, the expression of GluR1 protein in the ACC of the model group was increased compared to that of the control group (*P* < 0.05). A trend toward a reduced expression of GluR1 protein in the ACC of the EA group was observed (*P* > 0.05).

### 3.6. The Effect of ZIP on Allodynia and Anxiety-Like Behaviors

**Figure 7:**
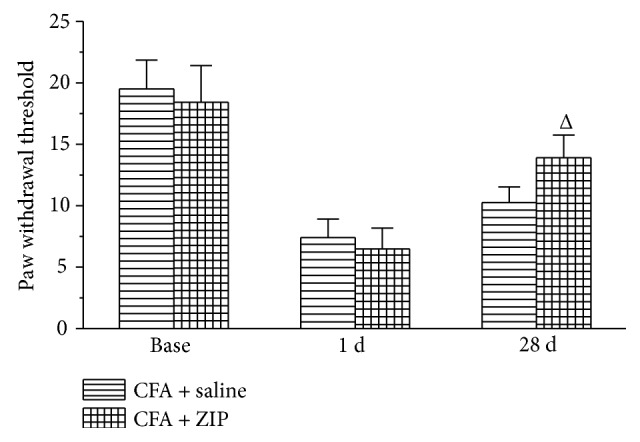
The effect of ZIP on the paw withdrawal threshold at different time points. The microinjection of ZIP into the ACC remarkably enhanced the paw withdrawal threshold. All data represent the mean ± SEM, *n* = 6. ^Δ^*P* < 0.05, compared to the CFA + saline.

**Figure 8:**
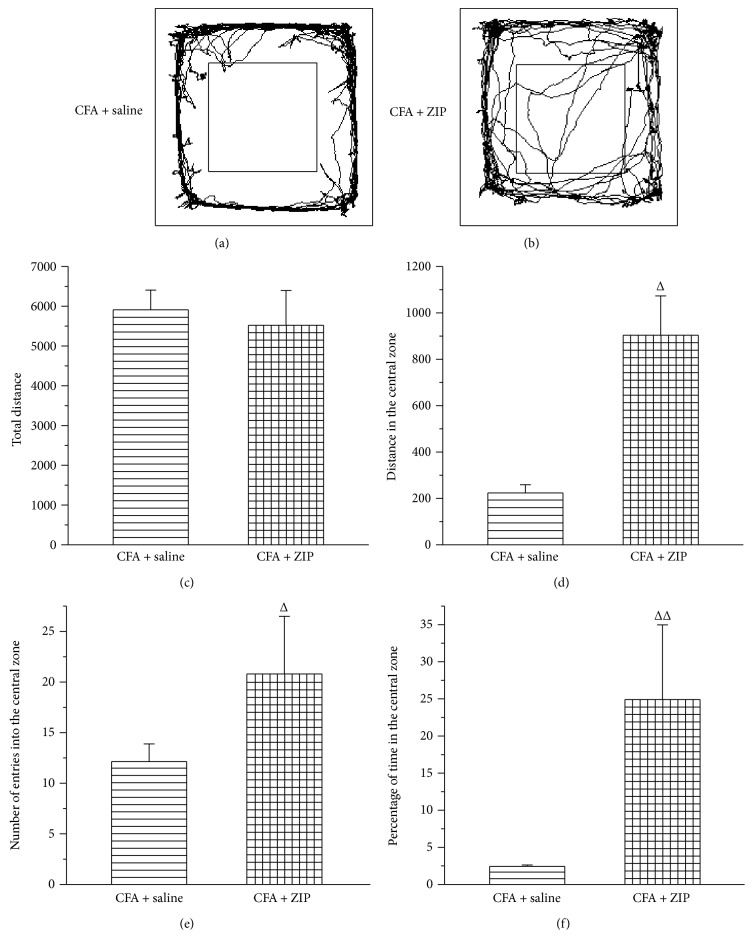
The effect of ZIP on chronic inflammatory pain-induced anxiety-like behaviors in the open-field test. (a, b) The trajectories of rats in the open-field test of CFA + saline group and CFA + ZIP group in the open-field test on day 29 after CFA injection. The total distance traveled throughout the arena (c), distance in the central zone (d), the number of entries into the central zone (e), and percentage of time in the central zone (f) of the CFA + saline group and CFA + ZIP group. All data represent the mean ± SEM, *n* = 6. ^Δ^*P* < 0.05 and ^ΔΔ^*P* < 0.01, compared to the CFA + saline.

**Figure 9:**
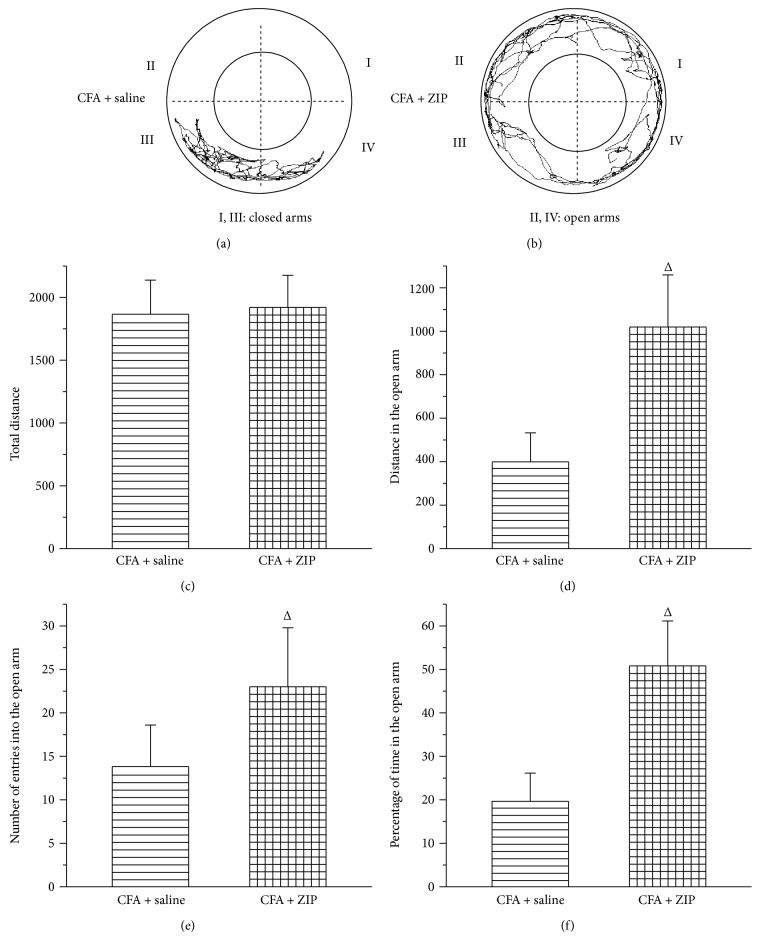
The effect of ZIP on chronic inflammatory pain-induced anxiety-like behaviors in the elevated zero maze. (a, b) The trajectories of rats in the CFA + saline group and CFA + ZIP group in the elevated zero maze on day 30 after CFA injection. The total distance traveled throughout the arms (c), distance traveled in the open arm (d), number of entries into the open arm (e), and percentage of time in the open arm (f). All data represent the mean ± SEM, *n* = 6. ^Δ^*P* < 0.05, compared to the CFA + saline.

To examine the contribution of PKMzeta to anxiety-like behavior, rats were injected with an inhibitor of PKMzeta (ZIP, 30 nmol/*μ*l, 1 *μ*l over 1 min) or 0.9% NS (1 *μ*l over 1 min) into the ACC once daily for three days beginning on day 28 after CFA injection. Forty-five min after the ZIP or NS injection, the rats underwent behavioral testing (PWT, open-field test, or elevated zero maze). The injection of ZIP into the ACC reduced the allodynia induced by the CFA injection (*P* < 0.05) (Figure 7). Moreover, injection of ZIP into the ACC also ameliorated the increase in anxiety-like behavior (*P* < 0.05) (Figures 8 and 9). In the open-field test, ZIP increased the distance traveled in the central zone, the number of entries into the central zone, and the percentage of time spent in the central zone (Figure 8). In the elevated zero maze, ZIP remarkably increased the distance in the open arms, the number of entries into the open arm, and the percentage of time spent in the open arm (Figure 9).

### 3.7. The Effect of ZIP on GluR1 Protein Expression in the ACC

**Figure 10:**
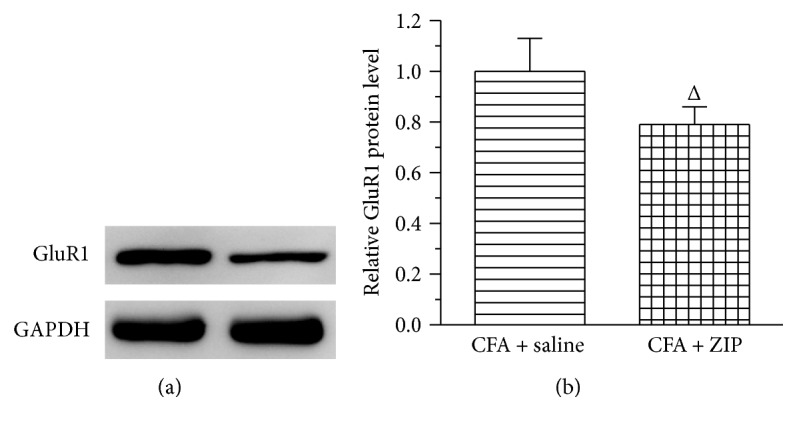
The effect of ZIP on GluR1 protein expression in the ACC. (a) Western blotting analysis revealed that ZIP significantly downregulated the protein expression of GluR1 on day 30 after CFA injection. (b) Quantification of (a) GluR1 protein normalized to the level of GAPDH. All data represent the mean ± SEM, *n* = 6. ^Δ^*P* < 0.05, compared to the CFA + saline.

We further observed whether ZIP might reduce the protein expression of GluR1 in the ACC on day 30 after CFA injection. As shown in Figure 10, ZIP significantly reduced the protein expression of GluR1 (*P* < 0.05).

## 4. Discussion

In the present study, chronic inflammatory pain induced by the injection of CFA was found to reduce the PWT and to increase anxiety-like behaviors. The expression of PKCzeta, p-PKCzeta (T560), PKMzeta, p-PKMzeta (T560), and GluR1 proteins in the ACC in the model group was higher than that of the control group. EA has analgesic and anxiolytic properties that counter the effects of chronic inflammatory pain. EA significantly inhibited the expression of p-PKMzeta (T560) protein in the ACC of CFA-injected rats and showed a trend to reduce the expression of PKCzeta, p-PKCzeta (T560), PKMzeta, and GluR1 protein. Furthermore, ZIP, a specific inhibitor of PKMzeta, increased the PWT, suppressed anxiety-like behaviors, and reversed the increase in GluR1 protein expression.

The symptoms of anxiety are very prevalent in chronic inflammatory pain patients, such as those with rheumatoid arthritis [30]. A growing body of literature has reported the development of anxiety-like behaviors in animal models of chronic inflammatory pain [6, 31, 32]. In this study, we found that rats with chronic inflammatory pain induced by CFA spent less distance in the central zone, had fewer number of entries into the central zone, and spent less percentage of time in the central zone of the open-field test compared to control rats. CFA-injected rats also traveled less distance in the open arm, had a reduced number of entries into the open arm, and spent less time in the open arm of the elevated zero maze compared to control rats.

EA, as a complementary and alternative medicine, has widely been used in many countries to treat various diseases, including pain. It is well known that pain has multiple dimensions, including sensory discrimination and affective motivation. A high percentage of chronic pain patients exhibit psychiatric disorders, such as anxiety and depressive disorders. Many recent studies have examined the analgesic effects of EA on pain sensory discrimination and the underlying mechanisms. The effect of EA on pain-related anxiety and depression is less well studied. In one clinical trial, patients receiving acupuncture experienced a significantly greater improvement in both the sensation of pain and pain-related anxiety than controls [33]. In an uncontrolled observation, acupuncture treatment had a more profound effect on the affective assessment than on the sensory assessment of pain [34]. Previous studies have indicated that EA can inhibit the negative affective response caused by CFA-induced inflammatory pain as assessed by conditioned place avoidance (CPA) [32]. The present study also demonstrates that EA treatment significantly suppresses CFA-induced anxiety-like behaviors as assessed by the open-field test and elevated zero maze. Additionally, it has been reported that EA attenuates anxiety-like behavior induced by chronic constriction injury [35] and L5 spinal nerve ligation [36] as assessed by the elevated plus maze. These and our data suggest that EA can regulate the affective disorders caused by chronic pain. However, the mechanism for these effects remains unclear.

The protein kinase C (PKC) family is an important group of genetic signaling modulators that have been identified as potential therapeutic targets for psychiatric disorders, cognitive dysfunction, and cancer. As such, PKC activators are considered candidates for cognitive-enhancement and antidementia therapeutics [37, 38]. Increases in PKC activity have been shown to increase post synaptic transport, and PKC inhibition has been shown to inhibit LTP, suggesting that PKC signaling might modulate long-term activity in neurons and synapses [39]. The PKC family can be divided into three subfamilies: classical PKC, novel PKC, and atypical PKC. PKCzeta and PKMzeta are atypical PKC isoforms. PKMzeta is an autonomously active atypical PKCzeta that is proposed to be a factor in the central nervous system plasticity, involved in chronic pain and psychiatric disorders [40, 41]. It has been reported that PKMzeta and its phosphorylated form were reported to be persistently increased in the ACC in a nerve injury model in mice [25]. Other studies have indicated that p-PKMzeta is increased in the rostral ACC (rACC) and that rACC infusion of ZIP leads to a long-lasting reversal of chronic neuropathic pain in rats [42]. One study indicated that PKMzeta is responsible for the maintenance of peripheral inflammation-primed persistent postsurgical pain (PPSP) [43]. These results suggest that spinal PKMzeta plays an essential role in the maintenance of persistent pain by sustaining spinal nociceptive plasticity [41]. In the present study, we established the induction of chronic inflammatory pain by CFA and assessed the involvement of PKCzeta and PKMzeta. Our results showed that PKCzeta, p-PKCzeta, PKMzeta, and p-PKMzeta proteins in the ACC were significantly increased on day 30 following CFA injection and that ZIP microinjection into the ACC could reverse the CFA-induced reduction in PWT. These results were similar to those of Marchand et al. [26], who showed that posttreatment ZIP administration attenuated the mechanical hypersensitivity in rats with CFA-induced hind paw inflammation. It has also been demonstrated that the blockade of PKMzeta alleviates tonic pain-related aversion in mice with a peripheral nerve injury [44]. We also assessed the effect of ZIP microinjection into the ACC on anxiety-like behaviors in the open-field test and the elevated zero maze. ZIP increased the distance traveled in the central zone, the number of entries into the central zone, and the percentage of time in the central zone in the open-field test. In the zero maze, ZIP remarkably increased the distance traveled in the open arm, the number of entries into the open arm, and the percentage of time spent in the open arm. These results demonstrate that PKMzeta plays an important role in pain-related anxiety.

PKMzeta has been reported to interact with GluR1 to maintain LTP in the ACC [27], a key brain region involved in the affective components of pain [15, 45]. The blockade of PKMzeta significantly reduces the postsynaptic GluR1 insertion in ACC neurons following a peripheral nerve injury [25, 44, 46]. In this study, we found that ZIP, a specific inhibitor of PKMzeta, suppressed the protein expression of GluR1.

Regarding EA analgesia and its mechanism in treating inflammatory pain, several studies have focused on the analgesic effect of EA on the sensory-discriminative aspect of pain [18–21]; however, few studies have examined the effect and mechanisms of EA on the emotional-affective dimension of pain. A growing number of studies have indicated that EA can relieve pain-related anxiety [13, 14] through a mechanism that involves opioid receptor activation in the ACC [32]. However, the mechanisms underlying the effects of EA on inflammatory pain-related anxiety are not completely understood. In the present study, we showed that EA significantly alleviated the expression of p-PKMzeta protein in the ACC but does not affect the expression PKCzeta, p-PKCzeta, PKMzeta, and its downstream GluR1 protein.

In conclusion, the present study provides direct evidence that the PKCzeta/PKMzeta-GluR1 pathway plays a role in pain and pain-induced anxiety-like behavior. EA treatment both increases pain-related somatosensory behavior (PWT) and decreases pain-induced anxiety-like behaviors by suppressing PKMzeta activity in the ACC.
